# The Effects of Non-Pharmaceutical Interventions on COVID-19 Epidemic Growth Rate during Pre- and Post-Vaccination Period in Asian Countries

**DOI:** 10.3390/ijerph19031139

**Published:** 2022-01-20

**Authors:** Le Duc Huy, Nhi Thi Hong Nguyen, Phan Thanh Phuc, Chung-Chien Huang

**Affiliations:** 1 Health Management Training Institute, University of Medicine and Pharmacy, Hue University, Thua Thien Hue 491-20, Vietnam; leduchuy@hueuni.edu.vn (L.D.H.); nthnhi@hueuni.edu.vn (N.T.H.N.); 2 School of Health Care Administration, College of Management, Taipei Medical University, Taipei 106-75, Taiwan; phuc.pt@umc.edu.vn; 3 International Ph.D. Program in Biotech and Healthcare Management, College of Management, Taipei Medical University, Taipei 106-75, Taiwan; 4 Department of Social Work, University Medical Center, Ho Chi Minh City 70000, Vietnam; 5 Department of Long-Term Care & School of Gerontology Health Management, College of Nursing, Taipei Medical University, Taipei 110-31, Taiwan; 6 Department & School of Pharmacy, College of Pharmacy, Taipei Medical University, Taipei 110-31, Taiwan; 7 Quality Advisor, Department of Medical Quality, Taipei Municipal Wan Fang Hospital, Taipei Medical University, Taipei 116-96, Taiwan

**Keywords:** non-pharmaceutical interventions, Asia, COVID-19, vaccine, longitudinal analysis

## Abstract

There is little knowledge about how the influence of non-pharmaceutical interventions (NPIs) reduces the COVID-19 infection rate during the period of vaccine rollout. This study aimed to examine the effectiveness of NPIs on decreasing the epidemic growth of COVID-19 between before and after the vaccine rollout period among Asian countries. Our ecological study included observations from 30 Asian countries over the 20 weeks of the pre- and post-vaccination period. Data were extracted from the Oxford COVID-19 Government Response Tracker and other open databases. Longitudinal analysis was utilized to evaluate the impacts of public health responses and vaccines. The facial covering policy was the most effective intervention in the pre-vaccination period, followed by border control and testing policies. In the post-vaccination period, restrictions on gatherings and public transport closure both play a key role in reducing the epidemic growth rate. Vaccine coverage of 1–5%, 5–10%, 10–30%, and over 30% of the population was linked with an average reduction of 0.12%, 0.32%, 0.31%, and 0.59%, respectively. Our findings support the evidence that besides the vaccine increasingly contributing to pandemic control, the implementation of NPIs also plays a key role.

## 1. Introduction

The COVID-19 pandemic has been continuously raising the enormous burdens of morbidity and mortality, leading to numerous consequences for societies and economies on humans worldwide, with nearly 270 million cases recorded, and more than 5.3 million deaths as of 13 December 2021 [1]. In 2020, many Asian governments obtained achievements in fighting against the COVID-19 pandemic thanks to introducing timely and robust non-pharmaceutical interventions (NPIs) [2]. These common NPIs included containment and closure policies (e.g., restrictions on gathering, school closure, public transport closure), and health system policies (e.g., testing policy, contact tracing) [2,3,4]. However, Asian countries have been facing new challenges in 2021. The vaccination coverage in most Asian countries is low, and far from the target of community immunity [5,6] as a result of the lack of vaccine supply, and vaccine hesitancy [7,8]. Besides, the emerging novel variants led to the resurgence of outbreaks in Asian countries [9,10,11], and threatened countries’ achievements in the pandemic control. Hence, the NPIs are still major tools for Asian nations to cope with pandemic waves before achieving herd immunity [12].

As far as we know, previous studies had demonstrated the effectiveness of NPIs on mitigating the COVID-19 pandemic; however, they mostly focused on the first wave of the pandemic, with limited analysis of subsequent waves, or were mainly based on modeling studies [13,14]. Furthermore, most of them mainly focused on evaluating the effectiveness of NPIs during a short period (from 30 days to 3 months) [15,16,17,18], and lacked an investigation of the effects of NPIs in the post-vaccination period [19]. In addition, recent studies mentioned pandemic fatigue could occur after the long-term impact of NPIs [20,21], which might affect adherence to public health interventions, and decrease the effectiveness of NPIs [20]. Therefore, we conducted this study to investigate the effects of non-pharmaceutical interventions in Asian countries on the COVID-19 average daily growth rate before and after the vaccine rollout period.

## 2. Materials and Methods

### 2.1. Study Design

We carried out an ecological study comprising 30 Asian countries over the two periods before and after vaccine rollouts. Our inclusion criteria: (a) the countries that are in the Asian Pacific list defined by the United Nations [22], and (b) provide the available data of NPIs, (c) number of tests per thousand people on the Oxford COVID-19 Government Response Tracker (OxCGRT) [3], and (d) vaccine data on the global database of COVID-19 vaccination [23].

### 2.2. Variables

Our outcome of interest is the average daily growth rate (wADGR) of the cumulative weekly number of confirmed COVID-19 cases in a specific country or territory. The calculation of outcome was well developed in previous work [15]. The average daily growth rate is computed by the formula: ${\mathrm{wADGR}}_{t}=\sqrt[{7}]{\frac{N_{t}}{N_{t-1}}}$ – 1 (Supplementary Materials), where N_t_ and N_t−1_ denote the cumulative number of COVID-19 cases at the end of the given week t and week t − 1, respectively [15]. Information on the cumulative number of COVID-19 cases was extracted from a data repository sourced from OxCGRT [3].

The independent variables were the time-varying intensity of NPIs, and the vaccine coverage in each country. The data for the intensity of individual NPI was captured through OxCGRT [3]. We first focused on the stringency index, which is calculated using the containment and closure policies: school closure, workplace closure, public event canceling, public transport closure, stay at home requirements, restrictions on internal movement, border control (international travel controls), and public information campaign indicators. The stringency index is measured on a continuous scale from 0 to 100. Then, we also considered other health system policies, including testing policy, contact tracing, and facial covering. The levels of these NPIs are presented on an ordinal scale (e.g., 0 = no measures, 1 = recommend canceling, 2 = require canceling), and varied over time. We took into account the time delay of the government response, which is the time interval between the first day recording a COVID-19 case and the first day implementing the NPIs.

In terms of vaccine variables, we collected them from the global vaccine database [23], which included: percentage of people vaccinated with at least one dose, people fully vaccinated, and total vaccines per 100 people. In our study, vaccine coverage is defined as the percentage of the population vaccinated with at least one dose [24].

The following variables were used as control variables in our study: population size (number of people), population density (people per square kilometer), median age (years), gross domestic product (GDP (US dollar)), percentage of the total population living in urban areas, socio-demographic index, classification of country income (high income, upper middle income, low-middle income), mobility index (a measure of human mobility pattern), and universal health coverage (UHC) index (a measure of health service coverage). Table S9 provides the explanation of all variables and data sources. 

### 2.3. Study Periods

To compare the effectiveness of NPIs on the wADGR in different periods across the countries, we extracted weekly data on the time-varying cumulative total cases, the intensity of NPIs, and accumulative vaccination data in each country. 

#### 2.3.1. The Pre-Vaccination Period

As the time of implementation of the NPIs were varied between the countries, the study period for analysis of the impact of NPIs began after the first containment and closures measure was implemented. We deleted Jordan in our population, since data on total cases were inconsistent (a smaller number of cumulative cases at a later time point). Finally, the study sample included 600 country–week observations over 30 Asian countries.

The strict level of NPIs was measured at the onset of the study period (week 0), and was followed up for 20 weeks. Since NPIs take time to contain the COVID-19 transmission, the wADGR was recorded two weeks after the beginning of study periods as the maximum incubation period for COVID-19 [25]. 

#### 2.3.2. Post-Vaccination Period 

We added the vaccination data in our model to explore the effects of NPIs on the wADGR after the vaccine rollout period.

The study period starts when the vaccine data are first recorded in each country (week 0), and is followed up for 20 weeks. We dropped China and Kazakhstan from our study population because China had a large number of missing values of vaccine coverage in a long period, whereas Kazakhstan provided inconsistent data on total cases during this period. Finally, 28 countries, which consisted of 560 country–week observations, were included in the study period.

Since the vaccine takes at least 14 days to have protective effects, we measured wADGR with a two-week lag regarding the vaccination data [26,27]. 

**Figure 1:**
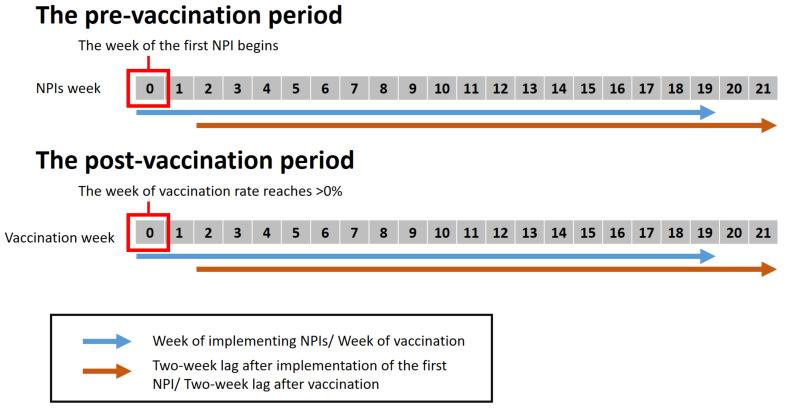
The definition of study periods.

Figure 1 presents how time points were defined in pre- and post- vaccination periods.

### 2.4. Statistical Analysis

We employed longitudinal analysis methods to evaluate the impact of NPIs and vaccine effects on epidemic growth rate in every study period. This approach was employed in previous studies to deal with longitudinal data on COVID-19 [15,17,28]. First, we used the linear mixed-effects model with a probit transformation of wADGR. This transformation was implemented because the probit is the best transformation approach for our proportional data (wADGR) that are highly skewed [29,30]. We then used the random intercepts and slopes model to account for the time-varying characteristic of an individual country. The forward selection approach, based on Bayesian information criterion (BIC), was used to develop the most paramount model. We begin with univariable analyses to select the independent variables showing statistical significance, and rank all of these variables in decreasing order according to BIC. Then, we add every independent variable into the model sequentially based on its rank, and remove insignificant ones. 

To facilitate the interpretation of NPI impacts, we utilized the multivariable beta regression generalized linear mixed model (mGLMM) with the probit link function, using wADGR as an outcome variable. Using this model, we can estimate the average marginal effects (AME) of NPIs, which represent the change in the wADGR as the result of changes in NPI intensity.

All analyses were carried out on R software. We built a linear mixed effect model using package lme4 with a maximum likelihood approach [31]. The generalized linear mixed model with a restriction maximum likelihood approach [32] was developed by the package, glmmTMB. We used the performance package to check each model’s AIC, BIC, and R square indexes [33].

## 3. Results

### 3.1. The Pre-Vaccination Period

Over the first study period in 2020, 2,539,514 people were reported infected with COVID-19 among 30 Asian nations (Table S10). The country with the highest number of cases per 100 people was Qatar (3.72 cases/100 people); the nation with the lowest number of cases per 100 people was Vietnam (0.00036/100 people).

**Figure 2:**
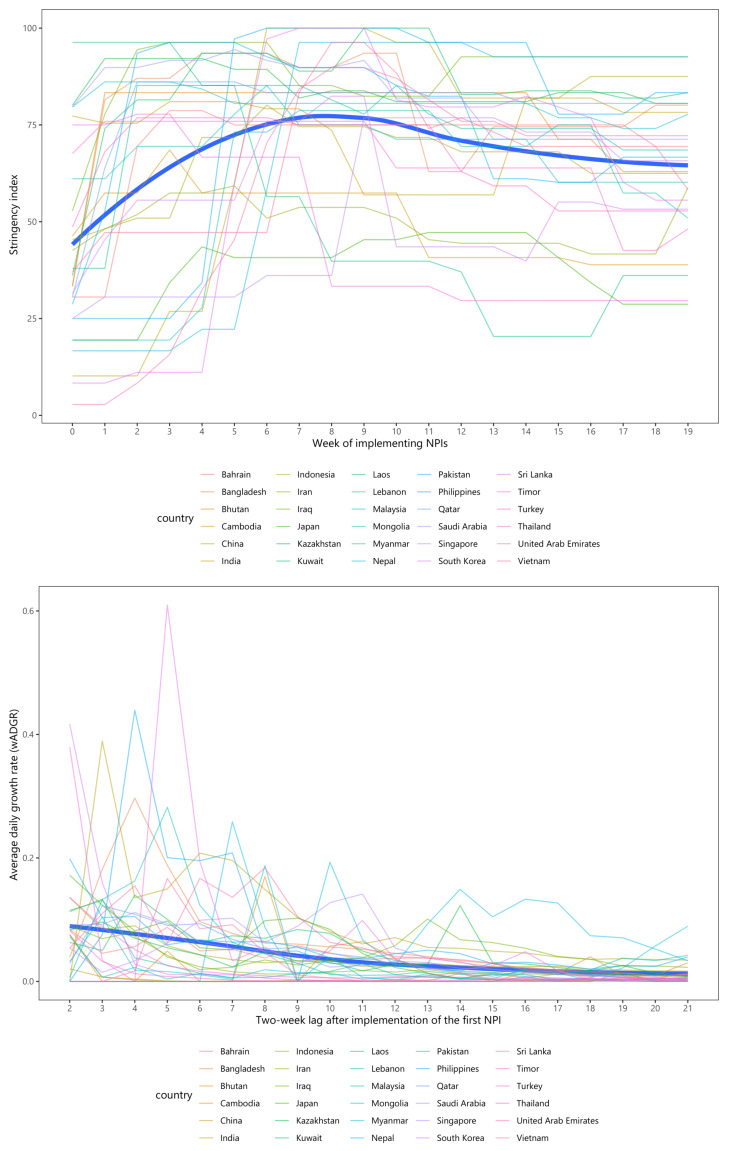
The evolution of stringency index and average daily growth rate (wADGR) over 20 weeks among Asian countries during the pre-vaccination period.

Figure 2 indicates the change in the stringency index and the wADGR throughout 20 weeks studied for each Asian country and overall. From Figure 2, the top panel shows that during the initial phase of the epidemic, the majority of Asian countries achieved high levels of NPI intensity, as expressed by the stringency index. These levels, in most cases, experienced an increasing fluctuation in NPI intensity for the first nine weeks, peaking at roughly 76 points, and slightly decreasing afterward. From the bottom panel in Figure 2, the two-week lagged wADGR witnessed a steep fluctuation in most countries, with a declining trend for the overall figure, reaching close zero values by the end of the study period. It was noticeable that Sri Lanka, where the wADGR in the fifth week reached a sharp peak of just over 0.6%, had a steep decrease afterward. India and Philippines reached peak wADGR at week 3 (at 0.39%) and week 4 (at 0.43%), respectively. Myanmar experienced a rising trend in the wADGR during the final three weeks of the study period, despite a minor fluctuation from week 18 onwards. 

**Figure 3:**
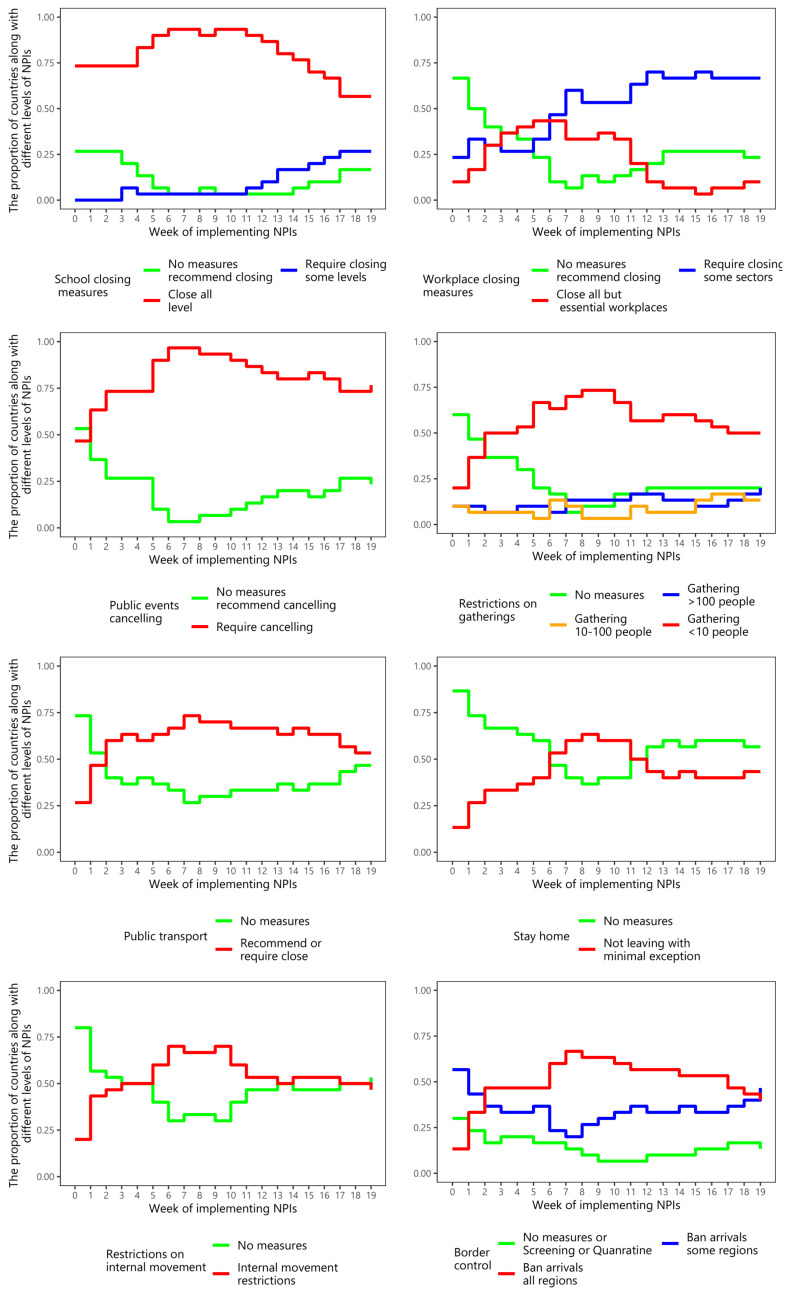
The proportion of countries implementing different intensities of NPIs over time among Asian countries during the pre-vaccination period.

Figure 3 shows the proportion of Asian nations with regard to implementing specific NPIs, and their intensities, during the pre-vaccination period. The NPIs, namely closing schools, and cancelling public events, were applied with the highest intensity level in most Asian nations. This was followed by restrictions on gatherings, and public transport closure.

**Figure 4:**
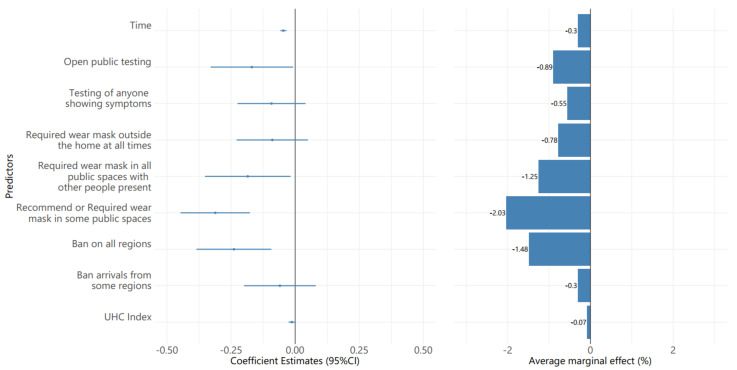
The effectiveness of NPIs on average daily growth rate (wADGR) among Asian countries in the pre-vaccination period.

Figure 4 demonstrates the effectiveness of NPIs on the wADGR of COVID-19 among Asian countries throughout the pre-vaccination period. The multivariable linear mixed effect model shows that testing policy, facial covering policy, and border control were significantly associated with a decrease in the probit transformation of wADGR. Of the control variables, the UHC index also showed a negative association with the probit wADGR, which means the higher the UHC index was, the lower the wADGR was. 

Based on the AME estimated from the multivariable generalized linear mixed model, we found that wearing masks was the most effective NPI to reduce the wADGR during this period. Altering the facial covering policy from “no recommended or required wear mask outside the home” to “recommended or required wear mask at some public space”, “required wear mask in all public spaces”, and “required wear mask all the time” was associated with reductions in the wADGR of 2.03%, 1.25%, and 0.78%, respectively. 

Border control policy was the second most effective NPI. Prohibiting all regions contributed to a 1.48% reduction of wADGR when compared to only screening or quarantining the arrivals. The widespread testing on the public or those with COVID-19 symptoms decreased wADGR by 1.73% and 0.62%, respectively, compared with a policy that only focused on people with symptoms, and who satisfied the specific criteria.

### 3.2. Post-Vaccination Period

Among 28 Asian countries, the proportions of countries with vaccine coverage and full vaccination less than 30% were around 70% and 82%, respectively (Table S11). Bhutan ranked first in terms of vaccination coverage, with 68.53%, followed by Mongolia (63.18%), and Qatar (59.57%). Iraq, Myanmar, and Bangladesh were nations with the lowest proportions of vaccine coverage, with 2.01%, 3.36%, and 3.5%, respectively. Regarding the full vaccination rate, the proportion of two-dose vaccination was the highest in Bhutan (60.78%), Mongolia (53.95%), and Qatar (50.5%), whereas the reverse pattern was true for Vietnam, Kuwait, and Iraq, with 0.38%, 0.88%, and 1.2%, respectively. 

**Figure 5:**
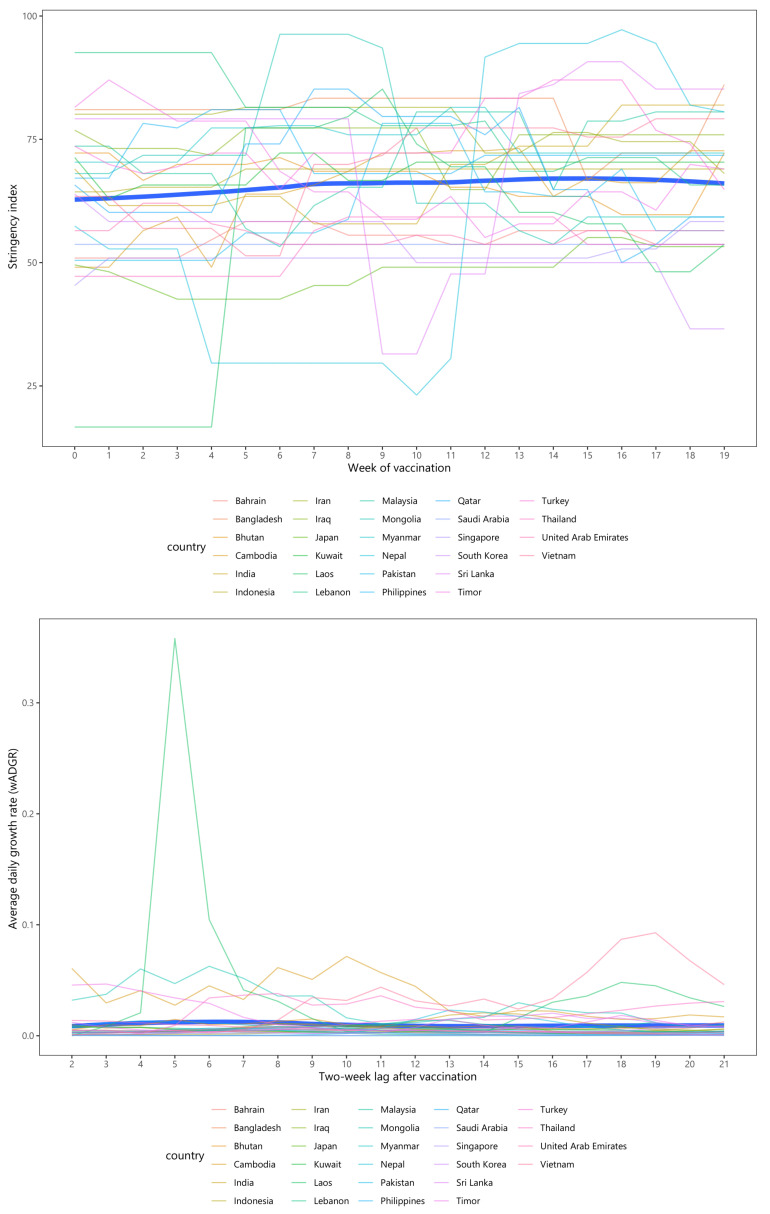
The evolution of stringency index and average daily growth rate (wADGR) among Asian countries during the post-vaccination period.

Figure 5 presents the changes in stringency index and wADGR over 20 weeks under the vaccine rollout scenarios in each Asian country, and overall. 

Asian countries kept stabilized in a high level of stringency policy (>60 points) over the study period. Though most countries experienced a lower wADGR in this period, several countries witnessed a surge in wADGR. The wADGR in Lao peaked in week five, and then dropped significantly over the next three weeks. Mongolia, Cambodia, Timor, and Vietnam also witnessed an increase in wADGR during this period, particularly Vietnam, which experienced a considerable increasing growth rate from the 16th week.

**Figure 6:**
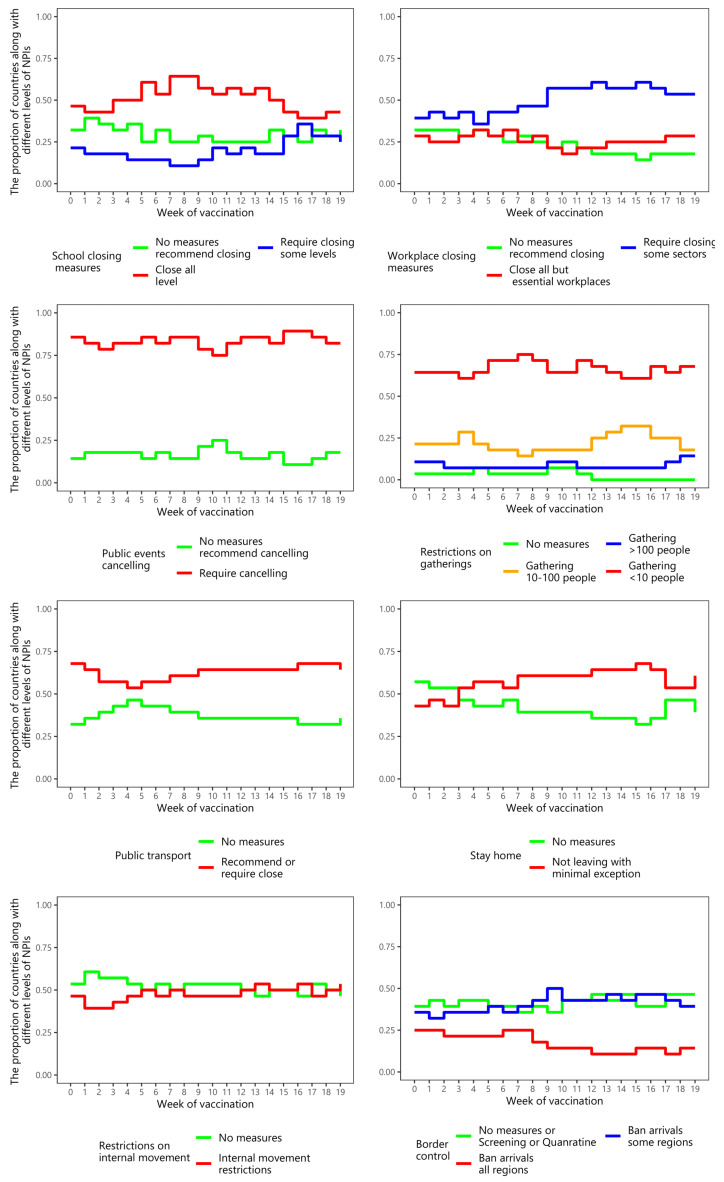
The proportion of countries implementing different intensities of NPIs over the 20 weeks among Asian countries during the post-vaccination period.

Figure 6 shows the percentage of countries with different levels of NPIs over the post-vaccination periods. Over time, the NPIs that a high percentage of countries employed at the high-intensity level were canceling public events and restrictions on gatherings, closing public transport, and requiring people to stay at home. Workplace closure and border control were applied at the middle level of intensity in most countries.

**Figure 7:**
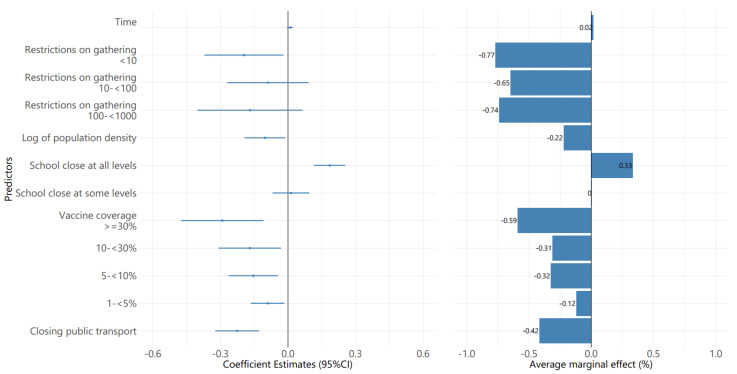
The effectiveness of NPIs on average daily growth rate (wADGR) among Asian countries under the vaccine rollout scenarios.

Figure 7 presents the effectiveness of NPIs under the vaccine rollout scenario in 2021. The results in this study period are different from the previous period in 2020. On the left panel of the multivariable linear mixed effect model results, we found significant predictors of changes in the probit_wADGR were restrictions on gathering, closing public transport, closing school, vaccine coverage, and a log of population density. 

The right panel of AME shows that restrictions on gathering have the highest effect on reducing the wADGR. Changes for restrictions on gathering from “no measure of restrictions on gathering” to restrictions on gatherings of <10 people, 10–100 people, and over 100 people were associated with decreasing the wADGR by 0.77%, 0.65%, and 0.74%, respectively. Regarding vaccine coverage, for the time that countries achieved 1–5%, 5–10%, 10–30%, and over 30%, the wADGR decreased by 0.12%, 0.32%, 0.31%, and 0.59%, respectively, compared to the period with below 1% vaccine coverage. The results indicated that closing public transport contributes to a lower wADGR of 0.42% compared to no measure of restrictions on public transport. In contrast, school closing at all levels increased wADGR by 0.33% compared to no measure. We also found a negative coefficient of the population density (log transformation), which indicated that the higher the population density, the lower the wADGR.

## 4. Discussion

To our knowledge, this is the first study to systematically investigate the effects of NPIs on the epidemic rate of COVID-19 in the pre- and post-vaccination period in Asian countries. Our finding highlighted that although the implementation of NPIs in the period of vaccine rollout has lower declining effects on epidemic rate compared with the initial phase of the pandemic, several NPIs are still considerably contributing to containing the widespread SARS-CoV2 virus. 

### 4.1. The Pre-Vaccination Period

We found that mask-wearing requirements, restrictions on international travelers, and testing policies significantly predict the reduction of the epidemic during the study period of 2020. 

There is evidence from cross-country studies that facial-covering policies demonstrated their effectiveness in containing the infection rate [15,19]. However, their effectiveness in the prevention of SARS-CoV transmission has been a controversial issue. Pozo Martin et al. showed that an earlier implementation of mask-wearing requirements led to a greater reduction in epidemic growth during the initial phase of the pandemic [15]. A recent meta-analysis study concluded that mask-wearing by the public is strongly recommended, especially when the widespread infection continues to grow, and physical distancing is unable to conduct [34]. However, scholars debated the effectiveness of mask-wearing by the public in countries where public events and gatherings at public spaces were strongly restricted [35]. 

We also observed that border control was effective during the outbreak stage of 2020; this result is consistent with those from previous studies [19,36]. In addition, recent studies showed that prohibitions on all international travel significantly decreased the reproduction number due to transmission from imported cases in Vietnam [37], and the peak number of cases in Kazakhstan [38]. 

Our findings showed evidence of a differential effect on epidemic growth when different levels of testing policy were implemented. This result is in line with previous studies [39]. In an analysis with data from 40 countries, Jeffrey et al. found that countries adopting broader testing have shown disease trajectory changes, and have the lowest COVID-19 mortality rate [39]. A typical example is South Korea, where performing a mass testing strategy was used as a key tool to detect the possible outbreaks, and flatten the epidemic curve [18]. However, this policy might result in an increased growth rate in the short term of the pandemic period [15,16]. 

With regards to the control variables, we found that, in countries with a higher UHC index, the wADGR tends to be lower. UHC index is the extent of the ability of a government to assure the provision of the essential health-related services people need in community settings [40]. The United Nations proposed that UHC is an essential foundation of an effective response to COVID-19 [41]. Furthermore, Yonghong et al. found that a higher UHC index was correlated with a higher reduction in mobility, which might increase the effectiveness of physical containment measures [42]. 

### 4.2. The Post-Vaccination Period

In comparison with the initial phase of the pandemic, our finding supports the evidence that NPIs have a smaller effect on decreasing the wADGR during the latter phase of the pandemic [15,43]. A possible explanation for this might be the influence of other factors on lowering the effects of public health measures, including the compliance of people with NPIs [44], pandemic fatigue [20], and the emergence of new variants [45]. However, NPIs keep playing a key role in preventing the widespread pandemic across Asian countries where vaccine rollout is still slow and has a low coverage rate. These results were also in line with the previous modeling studies [46,47,48]. A data-driven model study across 200 countries found that the combined effects of NPIs and vaccination might decrease 99% the COVID-19 burden [46].

Our finding found higher vaccination coverage was significantly associated with reducing epidemic growth rate. Liang et al. found that vaccine coverage was significantly linked to a reduction in case mortality rates after the percentage of the population vaccinated reached 8% [49]. Another study discovered that vaccination coverage could contribute more to preventing the spread of pandemic than vaccine efficacy [50].

Our study revealed that limitations on gatherings and public transport closure were both highly effective in reducing COVID-19 transmission under the vaccination scenarios. In a survey carried out in 130 countries, restrictions on gatherings had significant impacts on reducing the epidemic growth rate [19] during the latter phase of a pandemic. Liang et al. found that the effects of restrictions on gatherings on prolonging the double case were only consistent over a long period in the nations with a high level of government effectiveness [43]. Although the effectiveness of public transport closure was shown in studies during the earlier phase of the pandemic [16,17,51], we have not found that this measure was a predictor of the flattening of the epidemic growth under the vaccination period. Since the number of studies on the effects of NPIs in the post-vaccination period is limited, further study is needed to investigate the other factors related to the mechanisms behind this phenomenon. 

Surprisingly, the policy of school closure had the opposite effect on the reduction of infection rate. This finding is inconsistent with previous studies [43]. Liang et al. found the school closure was effective in prolonged COVID-19 case doubling time [43]. The opposite effect could reflect the new strategies of NPI implementation in the late pandemic or post-vaccination period [52]. In the countries witnessing a drop in new cases, the NPIs could be gradually relaxed to reduce the negative impacts on society and the economy, whereas countries with a continuous growth of new cases can keep implementing NPIs.

Our model also identified that population density was negatively associated with the epidemic growth rate of these control variables. This result is counterintuitive because the countries with higher population density tend to increase the contact probability, and raise the infection rate. Therefore, this result could be an artefact. A similar finding of an artefact was also observed in a previous study in which the countries with a higher percentage of an urban population led to a decrease in the epidemic rate [15]. 

Our study has some limitations. Firstly, all information of new cases was mainly based on the government report, and COVID-19 incidence might be underreported in some countries with limitations in their national health information systems or testing capability. Secondly, our model cannot cover all aspects or factors that might influence the growth rate of COVID-19 cases, including adherence to NPI policy, the quality of NPI implementation, and the influence of meteorological factors. Therefore, our results might have underestimated or overestimated the effectiveness of NPIs. Further studies are necessary when the data on these factors become available. In this study, available data on NPIs policies were mainly focused at the national level on the OxCGRT database; hence, regional policies were not included in our analysis.

Despite the above limitations, our studies have some significant contributions to the existing literature on the effectiveness of NPIs in combination with vaccination. Firstly, after integrating the vaccination data, we evaluated the effects of NPIs under vaccination scenarios; providing evidence to urge policymakers to pay more attention before mitigating the level of NPIs in the different contexts of vaccine coverage. Secondly, we utilized longitudinal data that can take into account the effects of NPIs in different time points within different countries. In addition, we extended the study period to 20 weeks to evaluate the stability of NPI effects on epidemic growth.

## 5. Conclusions

This study examined the effects of NPIs on the epidemic growth rate across 30 Asian countries during the pre- and post- vaccination period. The policies of facial coverings, testing, and border control considerably decreased the average daily growth rate in the weekly cumulative COVID-19 cases during the pre-vaccination period. In the latter period, restrictions on gatherings, and public transport closure were the most significant interventions to control the pandemic. Therefore, NPIs continue to play a significant role in curbing the COVID-19 pandemic, even with the implementation of vaccination. Our results make the implication that countries should be cautious in the mitigation of NPI intensity level before achieving the target immunization coverage rate.

## Data Availability

Data available in a publicly accessible repository. The data presented in this study are available in Supplementary Material Table S9: List of variables and data sources.

## Supplementary Materials

The following are available online at https://www.mdpi.com/article/10.3390/ijerph19031139/s1: Table S1. Univariate model results and sequence in which covariates were added in the forward selection multivariable linear mixed effect model during pre-vaccination period; Table S2. The final model of multivariable linear mixed effect model in pre-vaccination period; Table S3. The variance inflation factor (VIF) for covariates in the mLME during pre-vaccination period; Table S4. The final model of mGLM during the pre-vaccination period; Table S5. Univariate model results and sequence in which covariates were added in the forward selection multivariable linear mixed model during post-vaccination period; Table S6. The final model of multivariable linear mixed effect model in post-vaccination period; Table S7. The variance inflation factor (VIF) for covariates in the mLME during the post-vaccination period; Table S8. The final models for mGLM during the post-vaccination period; Table S9. List of variables and their data sources; Table S10. The number of COVID-19 cases per 100 people in the 20th week of pre-vaccination period across Asian countries; Table S11. The number of COVID-19 cases and vaccination rate in the 20th week of post-vaccination period across Asian countries; Figure S1. The residuals across all countries and over the time for the mLME during pre-vaccination period; Figure S2. The residuals in the intercepts and slopes across countries and in the between-country intercept and slope residuals for mLME during pre-vaccination period; Figure S3. The studentized residuals against the model’s fitted values during pre-vaccination period; Figure S4. The distribution of residuals across time point during pre-vaccination period; Figure S5. The residuals across all countries and over the time for the mLME during post-vaccination period; Figure S6. The residuals in the intercepts and slopes across countries and in the between-country intercept and slope residuals for mLME during post-vaccination period; Figure S7. The studentized residuals against the model’s fitted values during post-vaccination period; Figure S8. The distribution of residuals across time points during post-vaccination period.
