# Supplementary material for: The Effects of Non-Pharmaceutical Interventions on COVID-19 Epidemic Growth Rate during Pre- and Post-Vaccination Period in Asian Countries
Source: Int J Environ Res Public Health. 2022 Jan 20;19(3):1139. doi: 10.3390/ijerph19031139 (PMC8834794; doi:10.3390/ijerph19031139)
Supplement: Supplementary file 1 [file ijerph-19-01139-s001.zip › ijerph-1453458-supplementary.pdf]

Supplemental material

[ijerph-1453458]: The effects of non-pharmaceutical interventions on COVID-19 epidemic growth rate during pre- and post- vaccination period in Asian countries

Table of contents

1. Outcome Calculation 3
2. Model formulas 3
   2.1. Linear mixed effect model (LME) 3
   2.2. Generalized linear mixed model (GLM) 4
3. Model selection procedure and testing assumptions of model 6
   3.1. Pre-vaccination period 6
      3.1.1. Model selection of the multivariable linear mixed effect model (mLME) 6
      3.1.2. Testing the assumption of the mLME 8
      3.1.3. Multivariable generalized linear mixed model (mGLM) 12
   3.2. Post-vaccination period 13
      3.2.1. Model selection of the multivariable linear mixed effect model (mLME) 13
      3.2.2. Testing the assumption of the mLME 14
      3.2.3. Multivariable generalized linear mixed model (mGLM) 17
4. Data input and data sources 18
5. The supplement results regarding the number of COVID-19 cases and vaccine coverage rate 26
6. R Code 28
   6.1. List of variables used in the analysis 28
   6.2. Import R packages 29
   6.3. The R script in pre-vaccination period 29
   6.4. The R script for post-vaccination period 40

List of Tables

Table S1. Univariate model results and sequence in which covariate were added in the forward selection multivariable linear mixed model during pre-vaccination period. 6

Table S2. The final model of multivariable linear mixed effect model in pre-vaccination period. 8

Table S3. The variance inflation factor (VIF) for covariates in the mLME during pre-vaccination period 12

Table S4. The final model of mGLM during the pre-vaccination period. 12

Table S5. Univariate model results and sequence in which covariates were added in the forward selection multivariable linear mixed effect model (mLME) during post-vaccination period 13

Table S6. The final model of multivariable linear mixed effect model in post-vaccination period 14

Table S7. The variance inflation factor (VIF) for covariates in the mLME during the post-vaccination period. 17

Table S8. The final models for mGLM during the post-vaccination period. 17

Table S9. List of variables and data sources 18

Table S10. The number of COVID-19 cases per 100 people in the 20th week of pre-vaccination period across Asian countries 26

Table S11. The number of COVID-19 cases and vaccination rate in the 20th week of post-vaccination period across Asian countries 27

List of Figures

Figure S1. The residuals across all countries and over the time for the mLME during pre-vaccination period 9

Figure S2. The residuals in the intercepts and slopes across countries and in the between-country intercept and slope residuals for mLME during pre-vaccination period 10

Figure S3. The studentized residuals against the model's fitted values during pre-vaccination period 11

Figure S4. The distribution of residuals across time point during pre-vaccination period........... 11

Figure S5. The residuals across all countries and over the time for the mLME during post-vaccination period 14

Figure S6. The residuals in the intercepts and slopes across countries and in the between-country intercept and slope residuals for mLME during post-vaccination period................................... 15

Figure S7. The studentized residuals against the model's fitted values during post-vaccination period 16

Figure S8. The distribution of residuals across time point during post-vaccination period 16

1. Outcome Calculation

The formula of the weighted average daily growth rate in the cumulative number of weekly cases (wADGR).

$$N_t = (N_{t-1})(1 + wADGR_t)(1 + wADGR_t)(1 + wADGR_t)(1 + wADGR_t)(1 + wADGR_t)(1 + wADGR_t)(1 + wADGR_t)$$

$$N_t = (N_{t-1})(1 + wADGR_t)^7$$

$$wADGR_t = \sqrt[7]{\frac{N_t}{N_{t-1}}}\text{-1}$$

where N_t and N_{t-1} are the cumulative numbers of COVID-19 cases at the end of the given week t and week t-1 respectively.

For example: if there are 300 cases at the end of the 3rd week of the pandemic, and the cases at the end of the 4th week of the pandemic are 350, we have

$$wADGR_t = \sqrt[7]{\frac{350}{300}} - 1 = 0.0222$$

The average daily growth rate in 4th week is 2.22%.

2. Model formulas

2.1. Linear mixed effect model (LME)

$$\boldsymbol{Y_{i,t}} = \boldsymbol{\beta_0} + (\boldsymbol{\alpha} + \boldsymbol{\alpha_i})\boldsymbol{T_{i,t}} + \sum_{j=1}^{n}\boldsymbol{\beta_j X_{ij,t}} + \sum_{k=1}^{m}\boldsymbol{\theta_k P_{ik,t}} + (\boldsymbol{\delta_0 V_{i,t}}) + \boldsymbol{b_i} + \boldsymbol{\varepsilon_{i,t}} \quad \textbf{(1)}$$

Here the outcome variable $Y_{i,t}$ is the probit transformation of average daily growth rate (wADGR) in the country i on the week t.

The corresponding control variables that are significantly associated with outcome variable in univariate analysis are denoted by $X_{ij,t}$(j=1,…,n with n – number of selected control variables by the forward selection approach) their fixed effects are captured by β_j.

The NPIs variables that are significantly associated with outcome variable in univariate analysis are denoted by $P_{ik,t}$ (k=1,…,m with m – number of selected NPIs according to the forward selection approach) their fixed effect is captured by θ_k.

$V_{i,t}$ is the categorical variable for vaccination coverage of country i on week t. The effects of vaccination coverage is captured via the fixed effect δ_0.

The impacts of NPIs and vaccination coverage are the main predictors in our analysis.

The time since starting NPI implementation or vaccination is denoted by $T_{i,t}$ and its effect is captured via fixed effect α.

The random intercept model is used because it enables each country to have its own linear regression function with different intercept (corresponding to fixed intercept β_0). Country-specific random slopes for week $T_{i,t}$ might be important if the speed of average daily growth rate has varied by different weeks. Therefore, we extend the mixed models that allows the country-specific random slopes for week effect $T_{i,t}$ with a random effect α_i. Our model parameters are estimated via maximum likelihood (ML) approach.

The country-specific impact is captured via the random effect b_i ~ N (0,D). $\varepsilon_{i,t}$~ N (0, σ_e^2) is the country and week specific residual error.

D is the variance-covariance matrix for the intercept and slope pertaining to the outcome Y.

2.2. Generalized linear mixed model (GLM)

$$\mathbf{G(\mu_{i,t}) = \beta_0 + (\alpha + \alpha_i)T_{i,t} + \sum_{j=1}^{n} \beta_j X_{ij,t} + \sum_{k=1}^{m} \theta_k P_{ik,t} + (\delta_0 V_{i,t}) + b_i + \varepsilon_{i,t}} \quad \textbf{(2)}$$

Where link function G(·) refers to probit link function, that transforms the expectation of outcome, $\mu_{i,t} = E(Y_{i,t})$, to connect predictors in linear ways. Similar to the linear mixed effect model, the outcome variable $Y_{i,t}$ is the average daily growth rate (wADGR) in the country i on the week t.

The corresponding control variables that include significant outcome variable using univariate analysis are denoted by $X_{ij,t}$(j=1,…,n with n – number of selected control variables by the forward selection approach) their fixed effects are captured by β_j.

The NPIs variables include significant outcome variable using univariate analysis are denoted by $P_{ik,t}$ (k=1,…,m with m – number of selected NPIs according to the forward selection approach) their fixed effect is captured by θ_k.

$V_{i,t}$is the categorical variable for vaccination coverage of country i on week t. The effects of vaccination coverage is captured via the fixed effect δ_0.

The time since starting NPI implementation or vaccination is denoted by $T_{i,t}$ and its effect is captured via fixed effect α.

The country-specific impact is captured via the random effect b_i ~ N (0,D). $\varepsilon_{i,t}$~ N $(0,\sigma_e^2)$ is the country and week specific residual error.

D is the variance-covariance matrix for the intercept and slope pertaining to the outcome Y.

The random intercept and slope model is used. Hence, each country will have its own linear regression function with different intercept (corresponding to fixed intercept β_0) and country-specific random slopes for week effect $T_{i,t}$ with a random effect α_i. The generalized linear mixed model parameters are estimated via restricted maximum likelihood (REML) method.

3. Model selection procedure and testing assumptions of model

According to forward selection approach, the steps to select the final model include:

Step 1: we carried out the univariate analysis linear mixed effect models with time and each covariate (individual NPIs, vaccination coverage, control variables) as predictors

Step 2: We selected all the covariates which have significant value (p value <0.05) in the univariate analyses.

Step 3: We ranked the selected covariate in the decreasing order according to the goodness of fit of the univariate models as defined by Bayesian Information Criterion (BIC).

Step 4: We fitted a series of multivariable forward selection linear mixed models with time and inputted each covariate sequentially based on its rank. If an individual NPI was an insignificant predictor, it was dropped from the forward selection models.

Step 5: After achieving the final model for linear mixed effect model, we fitted the generalized linear mixed model with the similar covariates.

3.1. Pre-vaccination period

3.1.1. Model selection of the multivariable linear mixed effect model (mLME) during pre-vaccination period

After we carried out the univariate analysis, we identified the significant covariates. The covariates were ranked in decreasing order of good of fitness based on their BIC values. The results are presented in Table S1

Table S1. Univariate model results and sequence in which covariate were added in the forward selection multivariable linear mixed model during pre-vaccination period.

Rank	Covariates	Coefficient	p-values	BIC
1	Intercept	-1.28	0.00	516.71
	Ban arrivals from some regions	-0.05	0.48	
	Ban arrivals from all regions	-0.29	0.00	
	Time	-0.06	0.00	
2	Intercept	-1.23	0.00	519.26
	Require closing school at some levels	0.05	0.57	
	Require closing school at all levels	-0.21	0.00	
	Time	-0.06	0.00	
3	Intercept	-1.36	0.00	521.42

	Recommend or required wearing mask in some place	-0.34	0.00	
	Required wearing mask at all public spaces	-0.22	0.01	
	Required wearing mask outside the home at all time	-0.13	0.08	
	Time	-0.05	0.00	
4	Intercept	-1.37	0.00	522.53
	Require not leaving house	-0.15	0.00	
	Time	-0.06	0.00	
5	Intercept	-1.30	0.00	523.27
	Require closing workplace for some sectors	-0.16	0.01	
	Require closing workplace all	-0.22	0.00	
	Time	-0.06	0.00	
6	Intercept	-1.30	0.00	524.66
	Require canceling public events	-0.16	0.00	
	Time	-0.06	0.00	
7	Intercept	-0.24	0.55	524.86
	UHC index	-0.02	0.01	
	Time	-0.06	0.00	
8	Intercept	-0.48	0.17	525.76
	Median age	-0.03	0.01	
	Time	-0.06	0.00	
9	Intercept	-1.37	0.00	527.04
	Internal movement restrictions in place	-0.10	0.02	
	Time	-0.06	0.00	
10	Intercept	-1.81	0.00	527.58
	Limited contact tracing	0.59	0.02	
	Comprehensive contact tracing	0.29	0.21	
	Time	-0.06	0.00	
11	Intercept	-1.39	0.00	529.54
	Testing anyone showing COVID-19 symptoms	-0.09	0.21	
	Open public testing	-0.25	0.00	
	Time	-0.05	0.00	
12	Intercept	-1.30	0.00	531.25
	Restrictions on gatherings of between > 100	-0.04	0.67	
	Restrictions on gatherings of between 11 and 100 people	-0.15	0.10	
	Restrictions on gatherings of 10 people or less	-0.22	0.00	

	Time	-0.06	0.00	
13	Intercept	-1.30	0.00	534.57
	Upper middle income	-0.35	0.05	
	High income country	-0.06	0.73	
	Time	-0.06	0.00	

The table S2 indicates the multivariable linear mixed effect model (mLME) which includes the significant covariates selected from the forward selection during the pre-vaccination.

Table S2. The final model of multivariable linear mixed effect model in pre-vaccination period

Covariates	Coefficient	p values	BIC
Intercept	-0.32	0.46	524.6
UHC index	-0.01	0.04	
Ban arrivals from some regions	-0.06	0.40	
Ban on all regions	-0.24	0.00	
Recommend or Required wear mask in some public spaces	-0.31	0.00	
Required wear mask in all public spaces with other people present	-0.18	0.03	
Required wear mask outside the home at all times	-0.09	0.21	
Testing of anyone showing symptoms	-0.09	0.17	
Open public testing	-0.17	0.04	
Time	-0.05	0.00	

3.1.2. Testing the assumption of the mLME during pre-vaccination period

- Normality in the distribution of the residuals

The figure S1 presents the residuals across all countries and over the time.

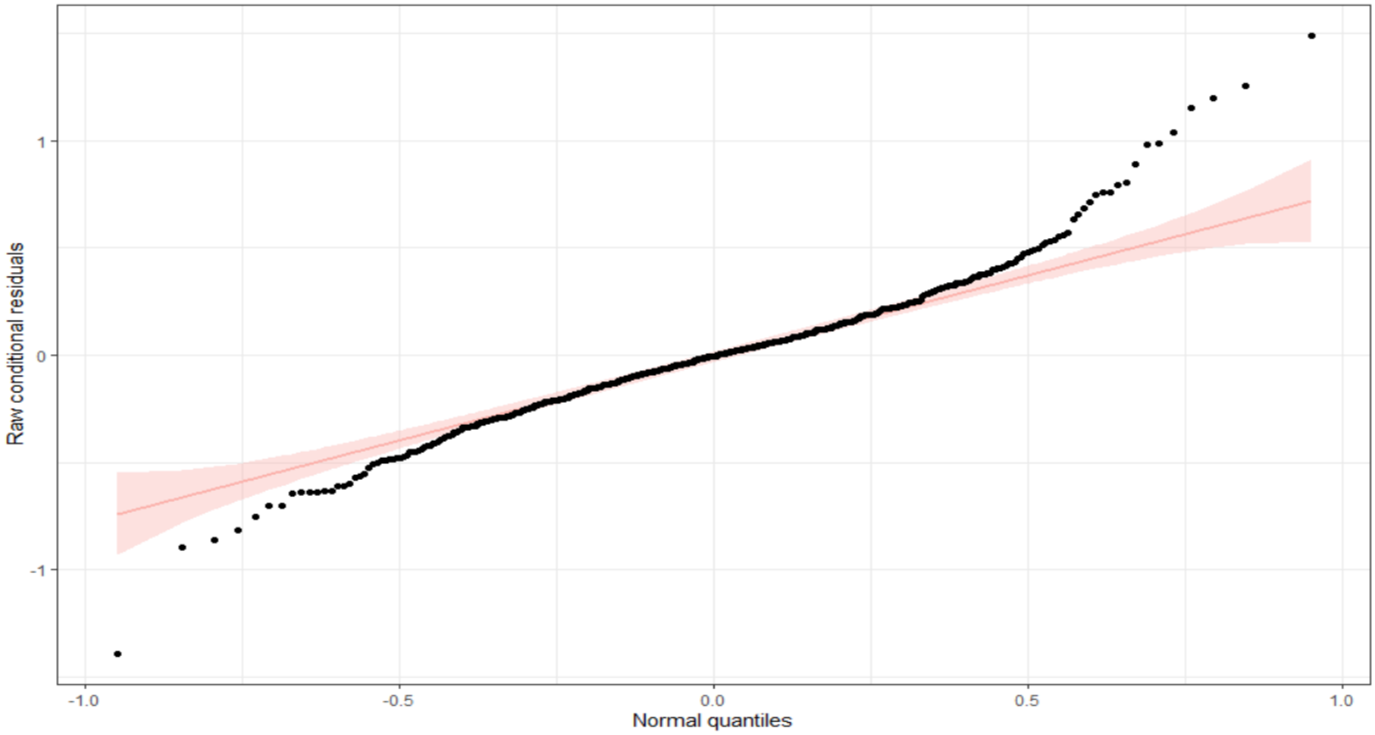

Figure S1. The residuals across all countries and over the time for the mLME during pre-vaccination period

The figure S2 shows the residuals in the intercepts and slopes across countries and in the between-country intercept and slope residuals in the pre-vaccination period.

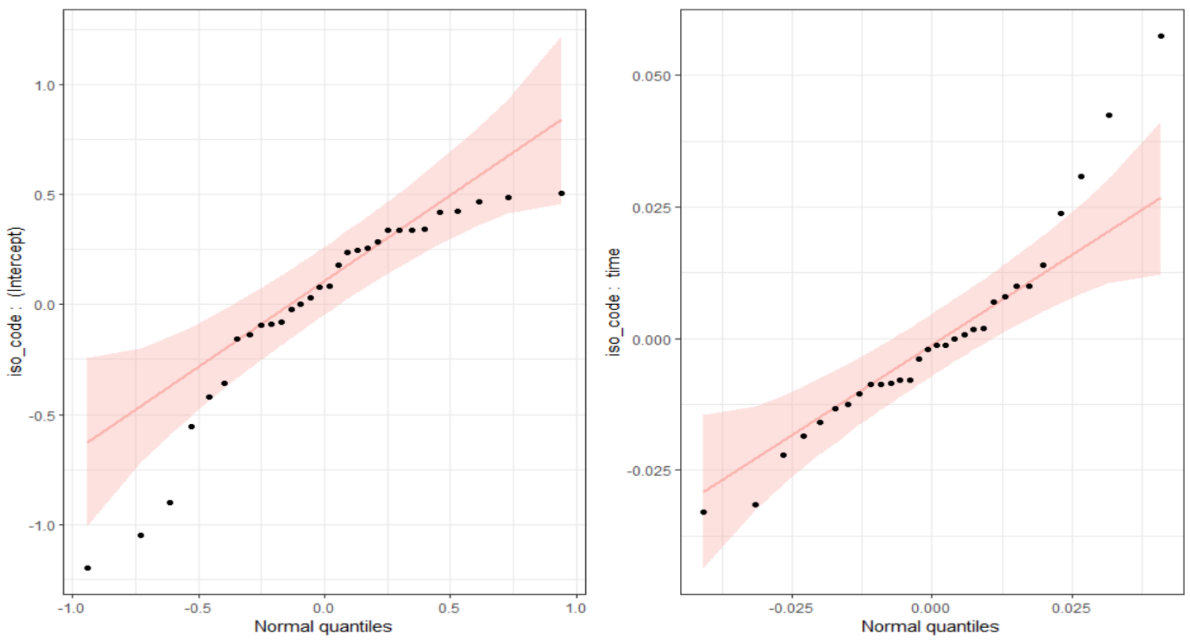

Figure S2. The residuals in the intercepts and slopes across countries and in the between-country intercept and slope residuals for mLME during pre-vaccination period

From Figure S1, S2 the distribution of these residuals can be considered approximately normal.

- Homoscedasticity in the distribution of the residuals

Figure S3 shows the studentized residuals against the model's fitted values. We can see an approximately constant variance of these residuals around zero across the predicted values of outcome.

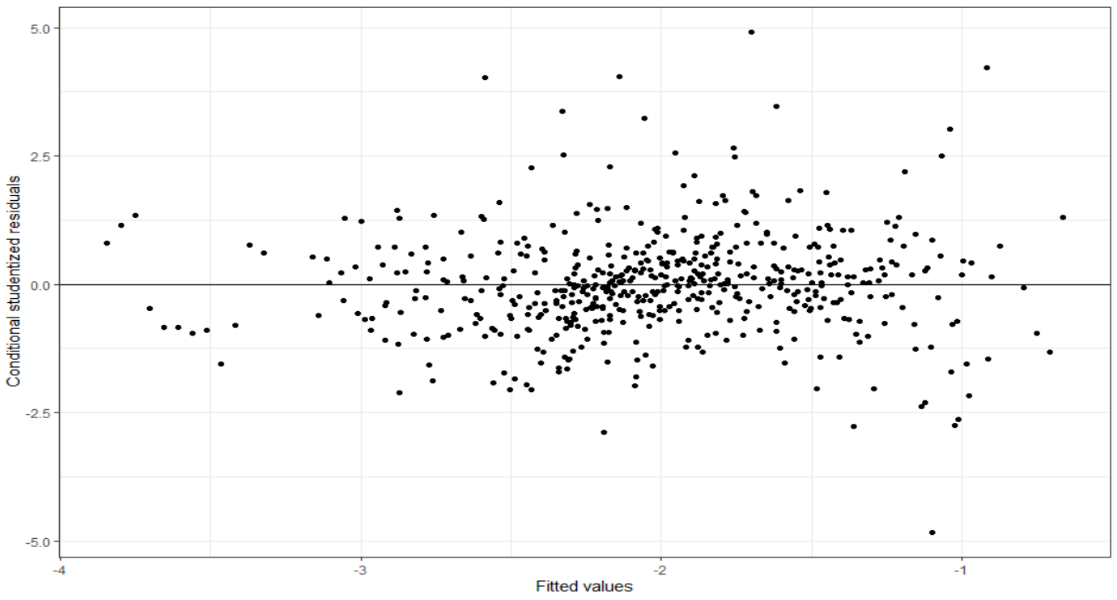

Figure S3. The studentized residuals against the model's fitted values during pre-vaccination period

Figure S4 presents the distribution of residuals across time points, showing approximately constant variance over the time.

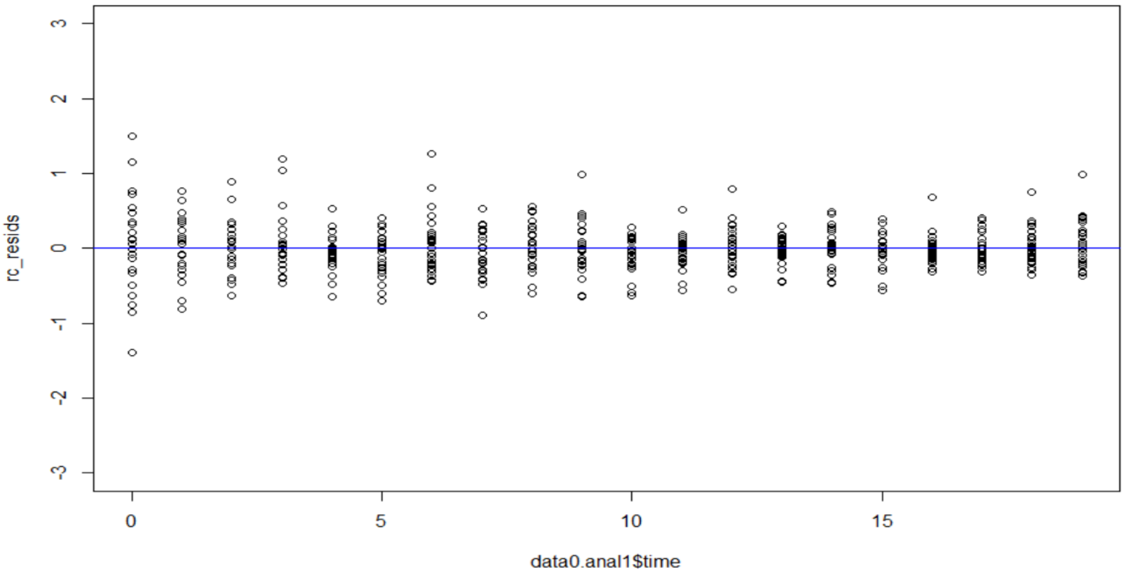

Figure S4. The distribution of residuals across time point during pre-vaccination period

- Low collinearity between predictors

Table S3 indicates the variance inflation factor (VIF) for covariates in the mLME. VIF values are about 1 which means lack of strong collinearity.

Table S3. The variance inflation factor (VIF) for covariates in the mLME during pre-vaccination period

Independent variables	VIF
UHC index	1.08
Border control	1.05
Facial coverings	1.21
Testing policy	1.16
Time	1.20

3.1.3. Multivariable generalized linear mixed model (mGLM)

We utilized a multivariable beta regression generalized linear mixed model (mGLM) with probit link function using the average daily growth rate (wADGR) as the dependent variable. The restricted maximum likelihood estimate (REML) was employed to establish the model.

To interpret the model results, we used the estimation of Average Marginal Effects (AME) to measure the changes of wADGR in the different levels of intensity of each NPIs. The AME shows how a dependent variable changes when a specific independent variable changes. Other covariates are assumed to be constant.

According to the manual instruction of AME calculation in R [1], we calculate a marginal effect for each NPIs. The code to calculate the AME for each NPIs is presented in section 6. R Code. The table S4 presents the final model of mGLM during the pre-vaccination period.

Table S4. The final model of mGLM during the pre-vaccination period.

Covariates	Coefficient	p values	Average Marginal Effects (AME)	BIC
Intercept	-0.65	0.02		-2619.9
UHC index	-0.01	0.04	-0.07%	
Ban arrivals from some regions	-0.03	0.63	-0.3%	
Ban on all regions	-0.16	0.02	-1.48%	
Recommend or Required wear mask in some public spaces	-0.24	0.00	-2.03%	
Required wear mask in all public spaces with other people present	-0.14	0.07	-1.25%	

Required wear mask outside the home at all times	-0.08	0.18	-0.78%	
Testing of anyone showing symptoms	-0.06	0.30	-0.55%	
Open public testing	-0.10	0.14	-0.89%	
Time	-0.03	0.00	-0.3%	

3.2. Post-vaccination period

3.2.1. Model selection of the multivariable linear mixed effect model (mLME) during post-vaccination period

Table S5 shows the results of the univariate analysis using mLME. The covariates were ranked in decreasing order of good of fitness based on their BIC values (only for covariate with statistical effects).

Table S5. Univariate model results and sequence in which covariates were added in the forward selection multivariable linear mixed effect model (mLME) during post-vaccination period

Rank	Covariates	Coefficient	p values	BIC
1	Intercept	-2.45	0.00	116.92
	Recommend or require closing public transport	-0.17	0.00	
	Time	0.00	0.90	
2	Intercept	-2.61	0.00	122.06
	Require school closing	-0.02	0.59	
	Require school closing all levels	0.11	0.00	
	Time	0.00	0.89	
3	Intercept	-2.01	0.00	127.83
	Log of population density	-0.11	0.01	
	Time	0.00	0.82	
4	Intercept	-2.30	0.00	133.52
	Restrictions on gatherings of between > 100	-0.30	0.01	
	Restrictions on gatherings of between 11 and 100 people	-0.22	0.01	
	Restrictions on gatherings of 10 people or less	-0.28	0.00	
	Time	0.00	0.91	
5	Intercept	-2.53	0.00	145.63
	Vaccine coverage (1-<5%)	-0.06	0.13	
	Vaccine coverage (5-<10%)	-0.12	0.05	
	Vaccine coverage (10-<30%)	-0.16	0.03	
	Vaccine coverage (>30%)	-0.26	0.01	
	Time	0.01	0.33	

The table S6 indicates the multivariable linear mixed effect model (mLME) which includes the significant covariates selected from the forward selection during the pre-vaccination.

Table S6. The final model of multivariable linear mixed effect model in post-vaccination period

Covariates	Coefficients	p value	BIC
Intercept	-7.12	0.00	123
Vaccine coverage (1-<5%)	-2.38	0.02	
Vaccine coverage (5-<10%)	-2.77	0.01	
Vaccine coverage (10-<30%)	-2.41	0.02	
Vaccine coverage (>=30%)	-3.13	0.00	
School close at some levels	0.32	0.75	
School close at all levels	5.23	0.00	
Log of population density	-2.31	0.03	
Restrictions on gathering 100-<1000	-1.41	0.16	
Restrictions on gathering 10-<100	-0.97	0.33	
Restrictions on gathering <10	-2.19	0.03	
Closing public transport	-4.55	0.00	
Time	1.37	0.18	

3.2.2. Testing the assumption of the mLME during post-vaccination period

- Normality in the distribution of the residuals

The figure S5 presents the residuals across all countries and over the time.

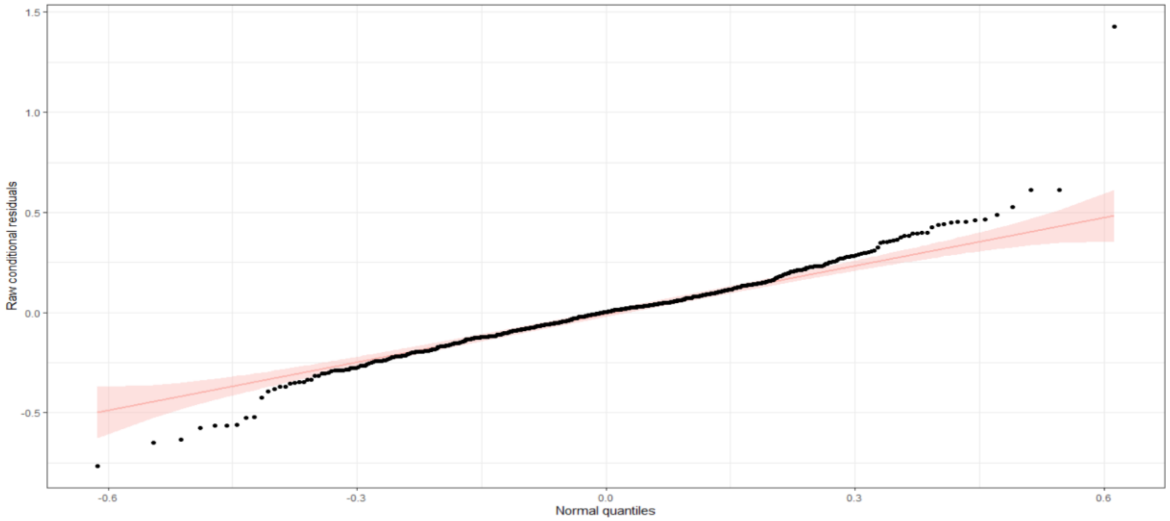

Figure S5. The residuals across all countries and over the time for the mLME during post-vaccination period

The figure S6 shows the residuals in the intercepts and slopes across countries and in the between-country intercept and slope residuals.

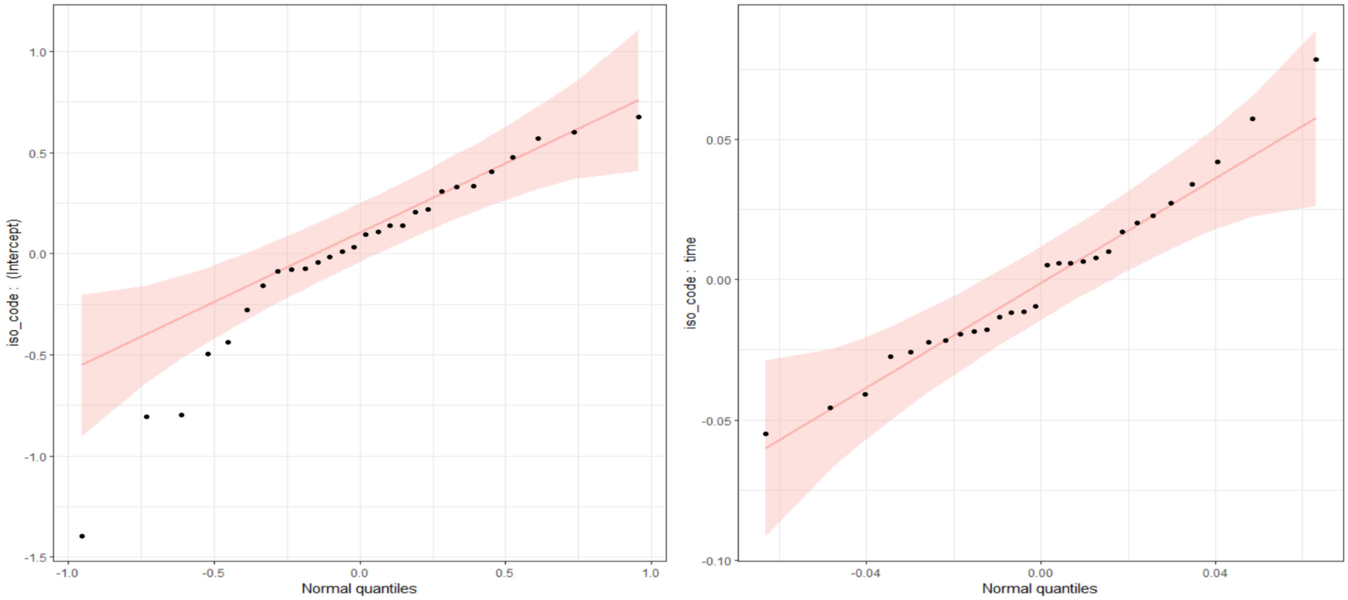

Figure S6. The residuals in the intercepts and slopes across countries and in the between-country intercept and slope residuals for mLME during post-vaccination period

From Figure S5, S6 the distribution of these residuals can be considered approximately normal.

- Homoscedasticity in the distribution of the residuals

Figure S7 shows the studentized residuals against the model's fitted values. We can see an approximately constant variance of these residuals around zero across the predicted values of outcome.

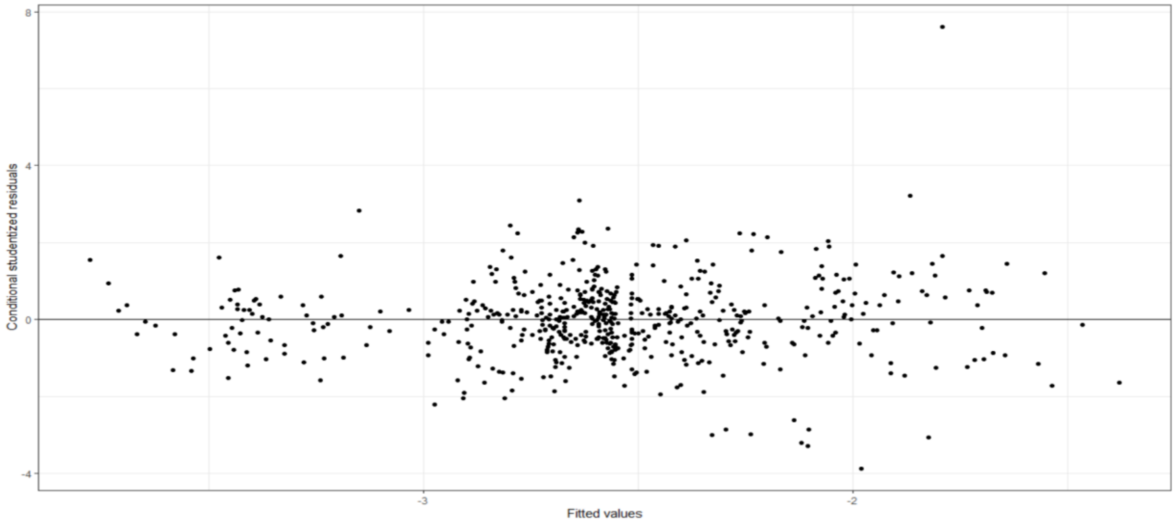

Figure S7. The studentized residuals against the model's fitted values during post-vaccination period

Figure S8 presents the distribution of residuals across time points, showing approximately constant variance over the time.

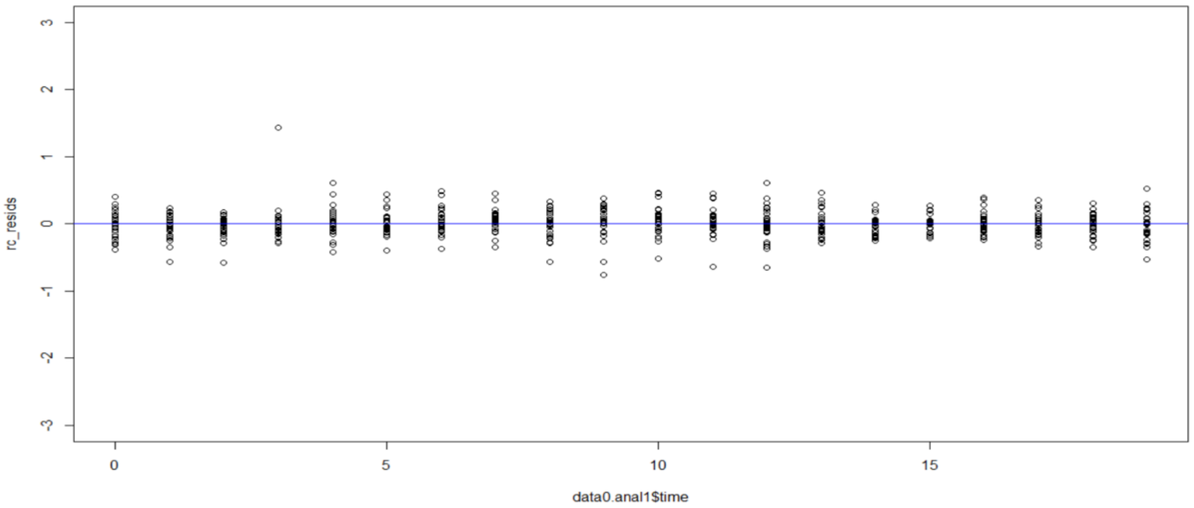

Figure S8. The distribution of residuals across time point during post-vaccination period

- Low collinearity between predictors

Table S7 indicates the variance inflation factor (VIF) for covariates in the mLME. VIF values are about 1 which means lack of strong collinearity.

Table S7. The variance inflation factor (VIF) for covariates in the mLME during the post-vaccination period.

Covariates	VIF
Vaccination coverage rate	1.34
Closing school	1.22
log of population density	1.02
Restriction gathering	1.56
Closing public transport	1.54
Time	1.25

3.2.3. Multivariable generalized linear mixed model (mGLM)

We utilized a multivariable beta regression generalized linear mixed model (mGLM) with probit link function using the average daily growth rate (wADGR) as the dependent variables. The restricted maximum likelihood estimate (REML) was employed to establish the model. Table S8 shows the final models for mGLM.

Table S8. The final models for mGLM during the post-vaccination period.

Covariates	Coefficients	p value	Average Marginal Effects (AME)	BIC
Intercept	-1.72	0.00		-4255.4
Closing public transport	-0.15	0.00	-0.42%	
Vaccine coverage (1-<5%)	-0.04	0.31	-0.12%	
Vaccine coverage (5-<10%)	-0.12	0.03	-0.32%	
Vaccine coverage (10-<30%)	-0.11	0.11	-0.31%	
Vaccine coverage (>=30%)	-0.24	0.01	-0.59%	
School close at some levels	0.00	0.97	0%	
School close at all levels	0.13	0.00	0.33%	
Log of population density	-0.08	0.02	-0.22%	
Restrictions on gathering 100-<1000	-0.22	0.07	-0.74%	
Restrictions on gathering 10-<100	-0.19	0.05	-0.65%	
Restrictions on gathering <10	-0.24	0.01	-0.77%	
Time	0.01	0.23	0.02%	

4. Data input and data sources

Table S9. List of variables and data sources

	Variables	Range value	Values recorded	Data source
1	C1_School closing	0 - No measures<br>1 - Recommend closing or all schools open with alterations resulting in significant differences compared to non-Covid-19 operations<br>2 - Require closing (only some levels or categories, eg just high school, or just public schools)<br>3 - Require closing all levels<br>Blank - no data	0 - No measures or recommend closing<br>2 - Require closing (only some levels or categories, e.g. just high school, or just public schools)<br>3 - Require closing all levels	Oxford database: The Oxford COVID-19 Government Response Tracker (OxCGRT)<br>Link: https://www.bsg.ox.ac.uk/research/research-projects/covid-19-government-response-tracker
2	C2_Workplace closing	0 - No measures<br>1 - Recommend closing (or recommend work from home) or all businesses open with alterations resulting in significant differences compared to non-Covid-19 operation	0 - No measures or recommend closing (or work from home)<br>2 - Require closing (or work from home) for some sectors or categories of workers<br>3 - Require closing (or work from home) all-but-essential workplaces (e.g. grocery stores, doctors)	

		2 - Require closing (or work from home) for some sectors or categories of workers<br>3 - Require closing (or work from home) for all-but-essential workplaces (eg grocery stores, doctors)<br>Blank - no data		
3	C3_Cancel public events	0 - No measures<br>1 - Recommend canceling<br>2 - Require canceling<br>Blank - no data	0 – No measures or recommend canceling<br>2 – Require canceling	
4	C4_Restrictions on gatherings	0 - No restrictions<br>1 - Restrictions on very large gatherings (the limit is above 1000 people)<br>2 - Restrictions on gatherings between 101-1000 people<br>3 - Restrictions on gatherings between 11-100 people<br>4 - Restrictions on gatherings of 10 people or less<br>Blank - no data	0 - No restrictions<br>1 - Restrictions on gatherings of between > 100<br>2 - Restrictions on gatherings of between 11 and 100 people<br>3 – Restrictions on gatherings of 10 people or less	

5	C5_Close public transport	0 - No measures<br>1 - Recommend closing (or significantly reducing volume/route/means of transport available)<br>2 - Require closing (or prohibiting most citizens from using it)<br>Blank - no data	0 – No measures<br>1 – Recommend closing (or significantly reduce volume/ route/ means of transport available) or require closing (or prohibit most citizens from using it)	
6	C6_Stay at home requirements	0 - No measures<br>1 - Recommend not leaving house<br>2 - Require not leaving house with exceptions for daily exercise, grocery shopping, and 'essential' trips<br>3 - Require not leaving house with minimal exceptions (eg allowed to leave once a week, or only one person can leave at a time, etc)<br>Blank - no data	0 – No measures or recommend not leaving house<br>1 - Require not leaving house with exceptions for daily exercise, grocery shopping, and 'essential' trips or require not leaving house with minimal exceptions (e.g. allowed to leave only once a week, or only one person can leave at a time, etc.)	

7	C7_Restrictions on internal movement	0 - No measures<br>1 - Recommend not to travel between regions/cities<br>2 - Internal movement restrictions in place<br>Blank - no data	0 – No measures or recommend not to travel between regions/ cities<br>2 – Internal movement restrictions in place	
8	C8_Internationa l travel controls	0 - No restrictions<br>1 - Screening arrivals<br>2 - Quarantine arrivals from some or all regions<br>3 - Ban arrivals from some regions<br>4 - Ban on all regions or total border closure<br>Blank - no data	0- No policy, Screening or Quarantine arrivals from some or all regions<br>1- Ban arrivals from some regions<br>2- Ban arrivals from all regions	
9	H1_Public information campaigns	0 - no Covid-19 public information campaign<br>1 - public officials urging caution about Covid-19<br>2- coordinated public information campaign (eg across traditional and social media)<br>Blank - no data	No applicable	

10	H2_Testing policy	0 - no testing policy<br>1 - only those who both (a) have symptoms AND (b) meet specific criteria (eg key workers, admitted to hospital, came into contact with a known case, returned from overseas)<br>2 - testing of anyone showing Covid-19 symptoms<br>3 - open public testing (eg "drive through" testing available to asymptomatic people)<br>Blank - no data	0-No testing policy or only those who have the symptoms<br>1- testing anyone showing COVID-19 symptoms<br>2- open public testing	
11	H3_Contact tracing	0 - no contact tracing<br>1 - limited contact tracing; not done for all cases<br>2 - comprehensive contact tracing; done for all identified cases	No applicable	

12	H6_Facial Coverings	0 - No policy<br>1 - Recommended<br>2 - Required in some specified shared/public spaces outside the home with other people present, or some situations when social distancing not possible<br>3 - Required in all shared/public spaces outside the home with other people present or all situations when social distancing not possible<br>4 - Required outside the home at all times regardless of location or presence of other people	0- No policy<br>1- Recommend or required in some specified shared/public spaces outside the home with other people present<br>2-Required at all shared/ public spaces<br>3- Required outside the home at all times regardless of location	
13	Stringency index	0 - 100	No applicable	
14	Cumulative number of confirmed cases of COVID-19	No applicable	No applicable	

15	Total number of SARS-COV-2 tests per thousand population	No applicable	No applicable	
16	Socio demographic Index	0-1	No applicable	Institute of Health Metrics and Evaluation www.healthdata.org/covid/datadownloads 2020
17	Mobility composite	No applicable	No applicable	
18	Universal health index	0-100	No applicable	Global Health Observatory indicator views https://apps.who.int/gho/data/node.imr 2019
19	% of total population living in urban areas	No applicable	No applicable	World Bank Open Data<br>Link: https://data.worldbank.org/
20	Population	No applicable	No applicable	
21	Population density	No applicable	No applicable	
22	Median age of population	No applicable	No applicable	
23	Classification of country's income	1-High income<br>2-Upper Middle Income<br>3-Lower Middle Income<br>4-Low Income	1-High income<br>2-Upper Middle Income<br>3-Low and Lower Middle Income (Low Middle Income)	

24	Total dose of vaccines administered	No applicable	No applicable	Oxford database: A global database of COVID-19 vaccinations<br><br>https://ourworldindata.org/covid-vaccinations<br>2021
25	Number of people vaccinated per 100 people in the total population of the country	0-100	0 : <1%<br>1 : 1-<5%<br>2 : 5-<10%<br>3 : 10-<30%<br>4 : >30%	
26	Number of people fully vaccinated per 100 people in the total population of the country	0-100	0 : <1%<br>1 : 1-<5%<br>2 : 5-<10%<br>3 : 10-<30%<br>4 : >30%	

5. The supplement results regarding the number of COVID-19 cases and vaccine coverage rate

Table S10. The number of COVID-19 cases per 100 people in the 20th week of pre-vaccination period across Asian countries

Country	Population	Total cases	Number of cases per 100 people
Qatar	2930524	109036	3.7207
Bahrain	1748295	36936	2.1127
Kuwait	4328553	59763	1.3807
Saudi Arabia	35340680	278835	0.7890
Singapore	5896684	41473	0.7033
Kazakhstan	18994958	107930	0.5682
United Arab Emirates	9991083	46133	0.4617
Iran	85028760	267061	0.3141
Turkey	85042736	236112	0.2776
Iraq	41179351	94693	0.2300
Bangladesh	166303494	229185	0.1378
Pakistan	225199929	267428	0.1188
Lebanon	6769151	2700	0.0399
Indonesia	276361788	100303	0.0363
India	1393409033	490401	0.0352
Philippines	111046910	34073	0.0307
Nepal	29674920	8605	0.0290
Malaysia	32776195	8600	0.0262
South Korea	51305184	12653	0.0247
Japan	126050796	17530	0.0139
Bhutan	779900	101	0.0130
Sri Lanka	21497306	1951	0.0091
Mongolia	3329282	293	0.0088
China	1444216102	83338	0.0058
Thailand	69950844	3310	0.0047
Timor	1343875	25	0.0019
Cambodia	16946446	240	0.0014
Myanmar	54806014	435	0.0008
Vietnam	98168829	349	0.0004
Laos	7379358	22	0.0003
Total	4427796980	2539514	0.0574

Table S11. The number of COVID-19 cases and vaccination rate in the 20th week of post-vaccination period across Asian countries

Country	Total cases	Percentage of population vaccinated at least 1 dose (vaccine coverage)	Percentage of population fully vaccinated
Bhutan	2566	68.53	60.78
Mongolia	141689	63.18	53.95
Qatar	222453	59.57	50.52
Saudi Arabia	509576	52.18	11.9
United Arab Emirates	550029	50.86	38.4
Bahrain	191018	46.11	33.67
Singapore	61940	35.82	27.41
South Korea	166722	30.44	11.46
Japan	812089	30.16	17.67
Timor	16402	27.93	13.5
Cambodia	46810	22.93	16.78
Malaysia	880782	22.68	12.92
Turkey	5263697	19.9	14.97
Kuwait	286046	18.99	0.88
Laos	7015	15.99	13.39
Thailand	381907	15.29	4.91
Lebanon	545226	13.67	6.67
India	28574350	12.51	3.16
Sri Lanka	225922	10.63	2.53
Philippines	1496328	9.16	3.86
Nepal	619635	8.43	2.47
Indonesia	1863031	6.43	4.05
Iran	3218860	4.79	1.93
Vietnam	94913	4.24	0.38
Pakistan	957371	4.13	1.38
Bangladesh	861150	3.5	2.57
Myanmar	145064	3.36	2.79
Iraq	1722328	2.01	1.2

6. R Code

6.1. List of variables used in the analysis

iso_code = The International Standard for country codes (Alpha-3 code)

country = name of states

time = Time of implementation NPIs in pre-vaccination dataset/ time of vaccination in post-vaccination dataset

week.case.eff = the average daily growth rate

probit.case = the average daily growth rate transformed using probit transformation

school.close.red = the intensity of closing school

work.close.red = the intensity of closing workplace

cancel.pubevent.red = the intensity of canceling public events

restric.gather.red = the intensity of restriction on gathering

close.transport.red = the intensity of closing public transportation

stay.home.red = the intensity of staying at home

restric.move.red = the intensity of restriction on internal movement

inter.travel.red = the intensity of border control

test.policy.red = the intensity of testing policy

contact.tracing = the intensity of contact tracing policy

wear.mask.red = the intensity of mask wearing policy

info.campaign = the intensity of public information campaign

pop_log = the log of population

pop_denlog = the log of population density

median_age = the median age of population

gdp.log = the log of GDP

test.thousand = the number of test per thousand residents

per_urban = percentage or population living in urban

uhc_index = Universal Health Coverage Index

healthworker_den = The Density of Health Care worker

incomegroup = the classification of country's income

sdi_index = Social Development Index

mobi.composite = mobility composite

pp.vac.100 = Percentage of population vaccinated at least one dose

ful.vac.100 = The percentage of population fully vaccinated

tt.vac.100 = The total number of vaccine administered

time.delay = Time delay of government response

6.2. Import R packages

```
install.packages("pacman")

library(pacman)

p_load(lme4,lmerTest ,performance, tidyverse, lubridate, lattice, devtools, VGAM, ggpubr,
plotly, knitr)
```

6.3. The R script in pre-vaccination period

```
# Set working directory

rm(list = ls())

wd <- "D:/covid_NPI"

dataset <- "D:/covid_NPI/dataset"

setwd(dataset)

load(file="prevac_data.RData")

# Develop function to extract the BIC values

library(performance)

bic <- function(model){

  fix2 <- performance(model)

  x<- as.character(substitute(model))

  fix2 <- fix2 %>%

    mutate( npi = x)
```

}

# Univariate analysis – NPIs

```
model.school.cls <- lmer(probit.case ~ school.close.red + time + (time|iso_code),
                 data= data0.anal, REML = F, lmerControl(optimizer = "bobyqa") )
summary(model.school.cls)
x1 <- bic(model.school.cls)

model.work.cls <- lmer(probit.case ~ work.close.red + time + (time|iso_code),
                 data= data0.anal, REML = F, lmerControl(optimizer = "bobyqa") )
summary(model.work.cls)
x2<- bic(model.work.cls)

model.pubevent <- lmer(probit.case ~ cancel.pubevent.red +  time + (time|iso_code),
                 data= data0.anal, REML = F, lmerControl(optimizer = "bobyqa") )
summary(model.pubevent)
x3<- bic(model.pubevent)

model.restr.gather <- lmer(probit.case ~ restric.gather.red +  time + (time|iso_code),
                 data= data0.anal, REML = F, lmerControl(optimizer = "bobyqa") )
summary(model.restr.gather)
x4 <- bic(model.restr.gather)

model.cls.trans <- lmer(probit.case ~ close.transport.red +  time + (time|iso_code),
                 data= data0.anal, REML = F, lmerControl(optimizer = "bobyqa") )
summary(model.cls.trans)
x5 <- bic(model.cls.trans)
```

```
model.stay.home <- lmer(probit.case ~ stay.home.red + time + (time|iso_code),
                    data= data0.anal, REML = F, lmerControl(optimizer = "bobyqa") )
summary(model.stay.home)
x6 <- bic(model.stay.home)

model.restr.move <- lmer(probit.case ~ restric.move.red +  time + (time|iso_code),
                    data= data0.anal, REML = F, lmerControl(optimizer = "bobyqa") )
summary(model.restr.move)
x7 <- bic(model.restr.move)

model.inter.trav <- lmer(probit.case ~ inter.travel.red + time + (time|iso_code),
                    data= data0.anal, REML = F, lmerControl(optimizer = "bobyqa") )
summary(model.inter.trav)
x8 <- bic(model.inter.trav)

model.test <- lmer(probit.case ~ test.policy.red +  time + (time|iso_code),
                    data= data0.anal, REML = F, lmerControl(optimizer = "bobyqa") )
summary(model.test)
x9 <- bic(model.test)

model.tracing <- lmer(probit.case ~ contact.tracing + time + (time|iso_code),
                    data= data0.anal, REML = F, lmerControl(optimizer = "bobyqa") )
summary(model.tracing)
x10 <- bic(model.tracing)

model.mask <- lmer(probit.case ~ wear.mask.red + time + (time|iso_code),
                    data= data0.anal, REML = F, lmerControl(optimizer = "bobyqa") )
summary(model.mask)
```

```
x11 <- bic(model.mask)

model.info <- lmer(probit.case ~ info.campaign + time + (time|iso_code),
                   data= data0.anal, REML = F, lmerControl(optimizer = "bobyqa") )
summary(model.info)
x12 <- bic(model.info)
```

# Univariate analysis – Vaccine variable and control variables

```
# number of population
model.pop <- lmer(probit.case ~ pop_log +
                        time + (time|iso_code),
                   data= data0.anal, REML = F, lmerControl(optimizer = "bobyqa") )
summary(model.pop)
y1 <- bic(model.pop)

#population density
model.popden <- lmer(probit.case ~ pop_denlog +  time + (time|iso_code),
                   data= data0.anal, REML = F, lmerControl(optimizer = "bobyqa") )
summary(model.popden)
y2 <- bic(model.popden)

# median age
model.age <- lmer(probit.case ~ median_age +  time + (time|iso_code),
                   data= data0.anal, REML = F, lmerControl(optimizer = "bobyqa") )
summary(model.age)
y3 <- bic(model.age)

# gdp
```

```
model.gdp <- lmer(probit.case ~ gdp.log +  time + (time|iso_code),
                  data= data0.anal, REML = F, lmerControl(optimizer = "bobyqa") )
summary(model.gdp)
y4 <- bic(model.gdp)

model.test1000 <- lmer(probit.case ~ test.thousand +  time + (time|iso_code),
                  data= data0.anal, REML = F, lmerControl(optimizer = "bobyqa") )
summary(model.test1000)
y5 <- bic(model.test1000)

# percentage of urban
model.per_urban <- lmer(probit.case ~ per_urban + time + (time|iso_code),
                    data= data0.anal, REML = F, lmerControl(optimizer = "bobyqa") )
summary(model.per_urban)
y6 <- bic(model.per_urban)

# uhc_index
model.uhc <- lmer(probit.case ~ uhc_index + time + (time|iso_code),
                    data= data0.anal, REML = F, lmerControl(optimizer = "bobyqa") )
summary(model.uhc)
y7 <- bic(model.uhc)

# health worker density
model.hworker <- lmer(probit.case ~ healthworker_den +   time + (time|iso_code),
                    data= data0.anal, REML = F, lmerControl(optimizer = "bobyqa") )
summary(model.hworker)
y8 <- bic(model.hworker)
```

```
#Income group
model.income <- lmer(probit.case ~ incomegroup +   time + (time|iso_code),
                    data= data0.anal, REML = F, lmerControl(optimizer = "bobyqa") )
summary(model.income)
y9 <- bic(model.income)

# SDI
model.sdi <- lmer(probit.case ~ sdi_index + time + (time|iso_code),
                    data= data0.anal, REML = F, lmerControl(optimizer = "bobyqa") )
summary(model.sdi)
y10 <- bic(model.sdi)

# mobi.composite
model.mobi <- lmer(probit.case ~ mobi.composite + time + (time|iso_code),
                    data= data0.anal, REML = F, lmerControl(optimizer = "bobyqa") )
summary(model.mobi)
y11 <- bic(model.mobi)

model.time.delay <- lmer(probit.case ~ time.delay + time + (time|iso_code),
                    data= data0.anal, REML = F, lmerControl(optimizer = "bobyqa") )
summary(model.time.delay)
y12 <- bic(model.time.delay)
```

# Select and rank the significant covariates

```
sig1 <- summary(model.school.cls)[["coefficients"]]
sig2 <- summary(model.work.cls)[["coefficients"]]
sig3 <- summary(model.pubevent)[["coefficients"]]
```

```r
sig4 <- summary(model.restr.gather)[["coefficients"]]
sig5 <- summary(model.stay.home)[["coefficients"]]
sig6 <- summary(model.restr.move)[["coefficients"]]
sig7 <- summary(model.inter.trav)[["coefficients"]]
sig8 <- summary(model.test)[["coefficients"]]
sig9 <- summary(model.tracing)[["coefficients"]]
sig10 <- summary(model.mask)[["coefficients"]]
sig11 <- summary(model.age)[["coefficients"]]
sig12 <- summary(model.uhc)[["coefficients"]]
sig13 <- summary(model.income)[["coefficients"]]

sig.covariate <-
as.data.frame(rbind(sig1,sig2,sig3,sig4,sig5,sig6,sig7,sig8,sig9,sig10,sig11,sig12,sig13))

bic.covariates <- rbind(x1, x2, x3, x4, x6, x7, x8, x9, x10, x11, y3, y7, y9)
```

# Linear mixed effect model

```r
model.mul <- lmer(probit.case ~ uhc_index  + inter.travel.red  + wear.mask.red +  test.policy.red
+ time  + (time|iso_code), data= data0.anal, REML = F, lmerControl(optimizer = "bobyqa") )
summary(model.mul)
performance(model.mul)
```

# Test assumption of linear mixed effect model

```r
devtools::install_github("goodekat/redres")
library(redres)

data0.anal1 <- data0.anal %>%  filter( is.na(uhc_index) ==F)
rc_resids <- compute_redres(model.mul)
pm_resids <- compute_redres(model.mul, type = "pearson_mar")
```

```
sc_resids <- compute_redres(model.mul, type = "std_cond")

resids <- data.frame(data0.anal1$iso_code, rc_resids, pm_resids, sc_resids)

plot_redres(model.mul, type = "std_cond")

plot_resqq(model.mul)

plot_ranef(model.mul)

plot(data0.anal1$time, rc_resids,ylim=c(-3,3))

abline(h=0, col="blue")

plot(data0.anal1$iso_code, sc_resids)

abline(h=c(0,2.96,-2.96), col="blue")

check_collinearity(model.mul))
```

# Generalized Linear Mixed Model

```
library(glmmTMB)

model.glm <- glmmTMB(week.case.eff ~ uhc_index  + inter.travel.red  + wear.mask.red +
test.policy.red + time + (time|iso_code), data = data0.anal, family = beta_family(link="probit"),
REML = TRUE)

summary(model.glm)
```

# Develop function to extract AME values

```
amex<- function(ame){
  x1 <- data.frame(list("AME" = ame*100, "factors" = as.character(substitute(ame))))
}
```

# Calculating the AME

```
coef_model <- summary(model.glm)$coefficients$cond[,1][-1]

coef <- as.data.frame(coef_model)


# AME time

AME.time <- coef_model[9] * mean(dnorm(predict(model.glm, type="link")))
```

```
z9 <- amex(AME.time)

# uhc index
AME.uhc_idx <- coef_model[1]* mean(dnorm(predict(model.glm, type="link")))
z1 <- amex(AME.uhc_idx)

# travel
trv0 <- predict(model.glm, newdata=data.frame(list("time"=data0.anal$time,
"uhc_index"=data0.anal$uhc_index,
"inter.travel.red"="0",
"wear.mask.red"=data0.anal$wear.mask.red,
"test.policy.red"=data0.anal$test.policy.red,
"iso_code"=data0.anal$iso_code) ), type = "response")

trv1 <- predict(model.glm, newdata=data.frame(list("time"=data0.anal$time,
"uhc_index"=data0.anal$uhc_index,
"inter.travel.red"="1",
"wear.mask.red"=data0.anal$wear.mask.red,
"test.policy.red"=data0.anal$test.policy.red,
"iso_code"=data0.anal$iso_code) ), type = "response")
trv2 <- predict(model.glm, newdata=data.frame(list("time"=data0.anal$time,
"uhc_index"=data0.anal$uhc_index,
"inter.travel.red"="2",
"wear.mask.red"=data0.anal$wear.mask.red,
"test.policy.red"=data0.anal$test.policy.red,
"iso_code"=data0.anal$iso_code) ), type = "response")

trv10 <- trv1 - trv0
```

```
trv20 <- trv2 - trv0

AME.trv10<-mean(trv10)
AME.trv20<-mean(trv20)

z2<-amex(AME.trv10)
z3<-amex(AME.trv20)
#wear.mask.red
mask0 <- predict(model.glm, newdata=data.frame(list("time"=data0.anal$time,
"uhc_index"=data0.anal$uhc_index,
"inter.travel.red"=data0.anal$inter.travel.red,
"wear.mask.red"="0",
"test.policy.red"=data0.anal$test.policy.red,
"iso_code"=data0.anal$iso_code) ), type = "response")
mask1 <- predict(model.glm, newdata=data.frame(list("time"=data0.anal$time,
"uhc_index"=data0.anal$uhc_index,
"inter.travel.red"=data0.anal$inter.travel.red,
"wear.mask.red"="1",
"test.policy.red"=data0.anal$test.policy.red,
"iso_code"=data0.anal$iso_code) ), type = "response")
mask2 <- predict(model.glm, newdata=data.frame(list("time"=data0.anal$time,
"uhc_index"=data0.anal$uhc_index,
"inter.travel.red"=data0.anal$inter.travel.red,
"wear.mask.red"="2",
"test.policy.red"=data0.anal$test.policy.red,
"iso_code"=data0.anal$iso_code) ), type = "response")
mask3 <- predict(model.glm, newdata=data.frame(list("time"=data0.anal$time,
"uhc_index"=data0.anal$uhc_index,
```

```
"inter.travel.red"=data0.anal$inter.travel.red,
"wear.mask.red"="3",
"test.policy.red"=data0.anal$test.policy.red,
"iso_code"=data0.anal$iso_code) ), type = "response")

mask10<-mask1-mask0
mask20<-mask2-mask0
mask30<-mask3-mask0

AME.mask10<-mean(mask10)
AME.mask20<-mean(mask20)
AME.mask30<-mean(mask30)

z4<-amex(AME.mask10)
z5<-amex(AME.mask20)
z6<-amex(AME.mask30)

# testing policy
test0<- predict(model.glm, newdata=data.frame(list("time"=data0.anal$time,
"uhc_index"=data0.anal$uhc_index,
"inter.travel.red"=data0.anal$inter.travel.red,
"wear.mask.red"=data0.anal$wear.mask.red,
"test.policy.red"="0",
"iso_code"=data0.anal$iso_code) ), type = "response")
test1<- predict(model.glm, newdata=data.frame(list("time"=data0.anal$time,
"uhc_index"=data0.anal$uhc_index,
"inter.travel.red"=data0.anal$inter.travel.red,
"wear.mask.red"=data0.anal$wear.mask.red,
```

```
"test.policy.red"="1",
"iso_code"=data0.anal$iso_code) ), type = "response")
test2<- predict(model.glm, newdata=data.frame(list("time"=data0.anal$time,
"uhc_index"=data0.anal$uhc_index,
"inter.travel.red"=data0.anal$inter.travel.red,
"wear.mask.red"=data0.anal$wear.mask.red,
"test.policy.red"="2",
"iso_code"=data0.anal$iso_code) ), type = "response")

test10<-test1-test0
test20<-test2-test0

AME.test10<-mean(test10)
AME.test20<-mean(test20)

z7<-amex(AME.test10)
z8<-amex(AME.test20)
```

6.4. The R script in post-vaccination period

We carried out the analysis with similar codes and added the vaccine variable.

# Linear mixed effect model

```
model.mul. vac <- lmer(probit.case ~   pp.vac.100+  school.close.red + pop_denlog +
restric.gather.red + close.transport.red + time  +  (time|iso_code),
                  data= data0.anal, REML = F, lmerControl(optimizer = "bobyqa") )
summary(model.mul.vac)
performance(model.mul.vac)
```

# Test assumption of LME model

```
data0.anal1 <- data0.anal %>%  filter( is.na(pp.vac.100) ==F) %>%
filter(is.na(close.transport.red) == F) %>% filter(is.na(school.close.red) == F) %>%
filter(is.na(restric.gather.red) == F)

rc_resids <- compute_redres(model.mul)

pm_resids <- compute_redres(model.mul, type = "pearson_mar")

sc_resids <- compute_redres(model.mul, type = "std_cond")

resids <- data.frame(data0.anal1$iso_code, rc_resids, pm_resids, sc_resids)

plot_redres(model.mul, type = "std_cond")

plot_resqq(model.mul)

plot_ranef(model.mul)

plot(data0.anal1$time, rc_resids,ylim=c(-3,3))

abline(h=0, col="blue")

plot(data0.anal1$iso_code, sc_resids)

abline(h=c(0,2.96,-2.96), col="blue")

check_collinearity(model.mul)
```

# Generalized Linear Mixed Model

```
library(glmmTMB)

model.glm <- glmmTMB(week.case.eff ~ close.transport.red + pp.vac.100 + school.close.red +
pop_denlog + restric.gather.red + time + (time|iso_code), data = data0.anal, family =
beta_family(link="probit"), REML = TRUE)

summary(model.glm)
```

Calculating the AME

We employed a similar method and codes to calculate the AME values for mGLM model for the post-vaccination period.
